# Spontaneous Resolution of Cervical Ligamentum Flavum Calcification: Imaging Evolution on Serial MRI

**DOI:** 10.7759/cureus.111277

**Published:** 2026-06-22

**Authors:** Alaa Al-Taie, Syeda Shabistan Intekhab, Syed Alam, Renan Ibrahem Adam

**Affiliations:** 1 Department of Radiology, Hamad General Hospital, Doha, QAT; 2 Department of Radiology, Hamad Medical Corporation, Doha, QAT; 3 College of Medicine, Qatar University, Doha, QAT; 4 Department of Musculoskeletal Radiology, Hamad Medical Corporation, Doha, QAT

**Keywords:** calcification, cervical spine, ligamentum flavum, magnetic resonance imaging, transient

## Abstract

Calcification of the ligamentum flavum (CLF) of the cervical spine is a rare but important cause of spinal cord compression. Although surgical decompression is the standard treatment for cases with cervical myelopathy, spontaneous resolution with conservative management has been reported. We present a case of a 69-year-old female with acute cervicobrachial pain and MRI findings of CLF at the C5 level, which resolved spontaneously on one-year follow-up MRI, supporting the potential for conservative management. Awareness of this phenomenon is important to avoid unnecessary surgical intervention.

## Introduction

Calcification of the ligamentum flavum (CLF) is characterized by the deposition of calcium crystals within the ligament. It is associated with metabolic and inflammatory conditions such as calcium pyrophosphate deposition disease [1,2]. It is usually observed in older individuals, particularly women over 60 years of age, and is more commonly seen in East Asian populations [3,4]. It has a low prevalence and is likely an underdiagnosed cause of spinal cord or nerve root compression [4].

CLF clinically presents as insidious or subacute cervical myelopathy due to posterior compression of the spinal cord by the calcified ligamentum flavum [1]. Approximately 20% of cases present with cervicobrachial neuralgia resulting from nerve root encroachment by the calcified ligamentum flavum [4]. Some patients have also presented with acute-onset neck pain with signs of inflammation such as fever, neck stiffness, and elevated inflammatory markers, mimicking infection [5]. This acute presentation is likely due to a crystal-induced inflammatory “flare” [5]. Such cases highlight the importance of recognizing CLF as a differential diagnosis for acute febrile neck pain to avoid unnecessary invasive interventions.

Surgical decompression is often curative for symptomatic CLF, whereas in some cases, the calcific masses may resolve spontaneously [4]. We present a case demonstrating the transient nature of cervical CLF, with spontaneous radiologic and clinical resolution documented on serial MRI, and discuss its implications for conservative management.

## Case presentation

A 69-year-old woman with a more than 10-year history of cervical and lumbar degenerative disc disease presented to the orthopedic clinic with severe neck pain radiating to the left upper limb, associated with occasional numbness. She also reported radicular pain in the distribution of the left C6 nerve root. There was no history of gait imbalance or distal neurological deficits.

On examination, vital signs were within normal limits. Inflammatory markers, including WBC, erythrocyte sedimentation rate, and CRP, were unremarkable. Cervical flexion and extension were painful, but no objective motor or sensory deficits were identified. Deep tendon reflexes were intact bilaterally. Hoffmann’s sign was negative.

Cervical X-rays showed straightening consistent with muscle spasm and mild degenerative changes at C5-C6, with no instability on dynamic views. MRI of the cervical spine showed moderate spondylosis with multilevel degenerative disc disease. A focal area of hypointense signal was seen on all sequences in the ligamentum flavum at the C5 vertebral level, suggestive of CLF. The calcified portion mildly indented the posterior aspect of the cervical spinal cord. No abnormal signal intensity was seen within the spinal cord (Figure 1).

**Figure 1:**
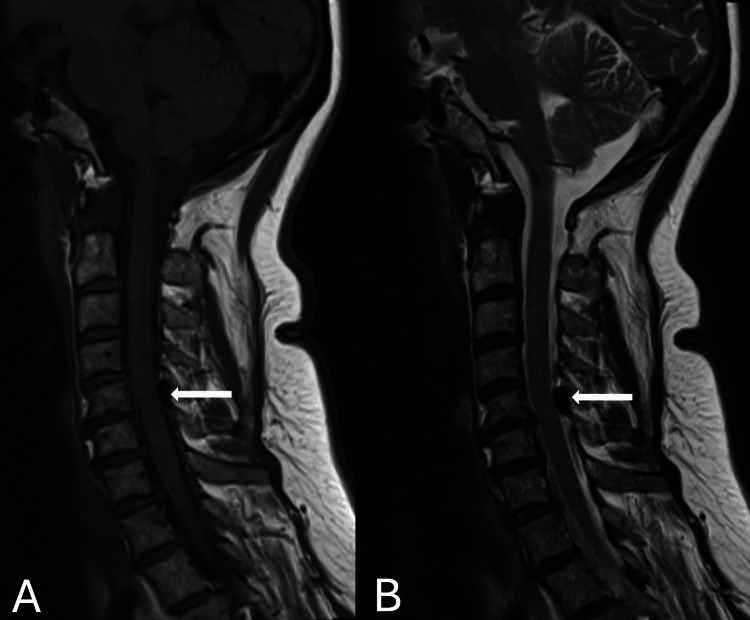
Sagittal MRI of the cervical spine. (A) T1- and (B) T2-weighted images showing a focal area of hypointense signal in the ligamentum flavum at the C5 level (arrows), consistent with CLF. CLF, calcification of the ligamentum flavum

The patient was managed conservatively with nonsteroidal anti-inflammatory drugs and physiotherapy. She reported gradual improvement in neck pain and numbness at subsequent follow-up visits. Repeat MRI of the cervical spine at one-year follow-up showed spontaneous resolution of the ligamentum flavum calcification (Figure 2). A previous MRI of the cervical spine from 2020 was available for comparison and demonstrated no CLF at that time, confirming interval development of CLF (Figure 3).

**Figure 2:**
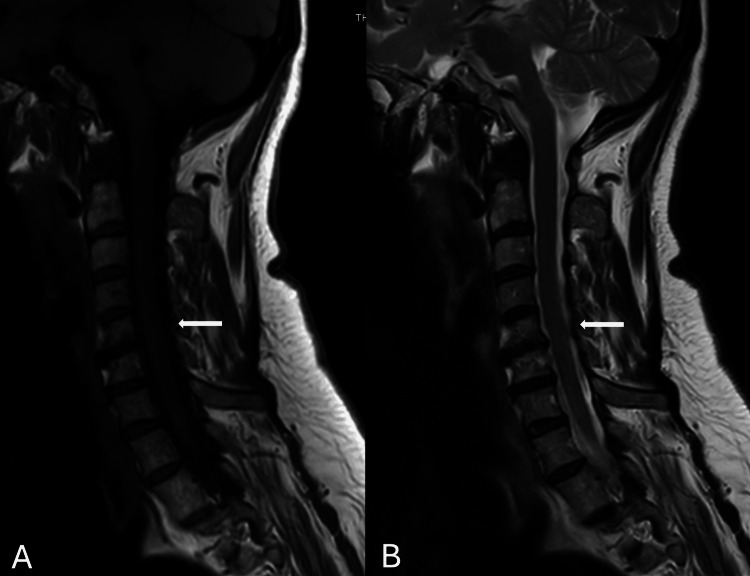
(A) T1- and (B) T2-weighted sagittal MRI images of the cervical spine one year later show spontaneous resolution of the calcified ligamentum flavum (white arrows).

**Figure 3:**
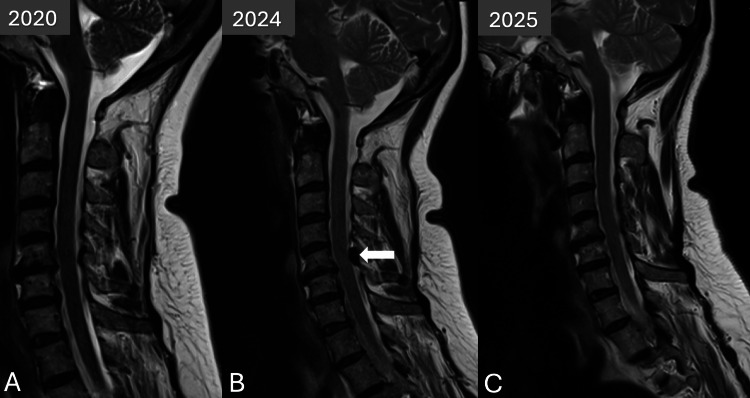
Serial sagittal T2-weighted MRI images of the cervical spine demonstrating the temporal evolution of ligamentum flavum calcification. (A) Initial MRI showing no calcification. (B) MRI four years later demonstrating a focal hypointense signal in the ligamentum flavum at the C5 level, consistent with new calcification. (C) MRI five years later confirming complete spontaneous resolution of the calcification.

## Discussion

Our case highlights an uncommon presentation of calcified ligamentum flavum with spontaneous resolution documented on serial MRI and symptomatic relief. This case contributes to the limited literature on the natural history and conservative management of CLF. The standard treatment for symptomatic CLF with myelopathy is surgical decompression; however, a conservative approach may be appropriate, particularly in cases where neurological deficits are absent or mild [4,6,7].

A limited number of prior case reports have described the resolution of ligamentum flavum calcifications with conservative management. Saliou et al. reported a patient with acute cervicobrachial radicular pain and a C7 ligamentum flavum calcific deposit that completely disappeared on follow-up CT after one week. The lesion showed signs of active inflammation and rim enhancement on MRI, suggesting that the inflammatory response may have facilitated resorption of the calcification. In the same patient, a chronic calcification at the C6 level, which showed no inflammatory changes on MRI, remained stable on follow-up. The authors therefore hypothesized that an acute inflammatory reaction can sometimes facilitate resorption of calcium deposits [8].

In another case, a middle-aged woman presented with acute febrile neck pain due to C5-C6 CLF, which subsided after treatment with colchicine. Cervical X-rays at two months showed near-complete disappearance of the calcification [5]. Most recently, Sarraj et al. reported a 72-year-old woman with cervical CLF at C3-C4 causing myelopathy, with complete spontaneous resolution on CT three months after symptom onset and confirmed on subsequent MRI. The patient was planned for surgical intervention, but the procedure was cancelled following radiographic resolution, underscoring the importance of repeat imaging before committing to surgery in patients who demonstrate clinical improvement [9]. Collectively, these cases demonstrate that spontaneous regression of ligamentum flavum calcification is possible and may potentially obviate the need for surgical intervention [5,8,9].

The implications of spontaneous CLF resolution are clinically significant. First, these findings suggest that an initial trial of conservative treatment is a reasonable approach in patients with mild or absent neurological deficits [8]. Published cases indicate that in acute CLF presentations with associated inflammation, nonsurgical therapy has led to symptom relief and lesion regression [5,8]. Second, recognizing the MRI features of acute inflammatory calcification may help identify lesions that have the potential to regress spontaneously [8,10]. Therefore, MRI not only aids in establishing the diagnosis but may also provide prognostic information regarding the likelihood of spontaneous resorption.

This case has some limitations. The one-year follow-up interval means the exact timing of resolution could not be determined. Additionally, as a single case report, generalizability remains limited. Ongoing documentation of similar cases could help refine diagnostic criteria, management strategies, and prognostic expectations for this uncommon and likely underdiagnosed condition [6,7,10].

## Conclusions

Cervical ligamentum flavum calcification is a rare and possibly underreported but important diagnosis to consider in patients presenting with spinal cord or nerve root compression. This case demonstrates that CLF can resolve spontaneously, as documented on serial MRI, and highlights that surgical intervention is not always necessary. Careful correlation of clinical presentation with MRI findings can guide the decision to pursue conservative management over surgical treatment.
